# Chapter 13: Mining Electronic Health Records in the Genomics Era

**DOI:** 10.1371/journal.pcbi.1002823

**Published:** 2012-12-27

**Authors:** Joshua C. Denny

**Affiliations:** Departments of Biomedical Informatics and Medicine, Vanderbilt University School of Medicine, Nashville, Tennessee, United States of America; Whitehead Institute, United States of America; University of Maryland, Baltimore County, United States of America

## Abstract

Abstract: The combination of improved genomic analysis methods, decreasing genotyping costs, and increasing computing resources has led to an explosion of clinical genomic knowledge in the last decade. Similarly, healthcare systems are increasingly adopting robust electronic health record (EHR) systems that not only can improve health care, but also contain a vast repository of disease and treatment data that could be mined for genomic research. Indeed, institutions are creating EHR-linked DNA biobanks to enable genomic and pharmacogenomic research, using EHR data for phenotypic information. However, EHRs are designed primarily for clinical care, not research, so reuse of clinical EHR data for research purposes can be challenging. Difficulties in use of EHR data include: data availability, missing data, incorrect data, and vast quantities of unstructured narrative text data. Structured information includes billing codes, most laboratory reports, and other variables such as physiologic measurements and demographic information. Significant information, however, remains locked within EHR narrative text documents, including clinical notes and certain categories of test results, such as pathology and radiology reports. For relatively rare observations, combinations of simple free-text searches and billing codes may prove adequate when followed by manual chart review. However, to extract the large cohorts necessary for genome-wide association studies, natural language processing methods to process narrative text data may be needed. Combinations of structured and unstructured textual data can be mined to generate high-validity collections of cases and controls for a given condition. Once high-quality cases and controls are identified, EHR-derived cases can be used for genomic discovery and validation. Since EHR data includes a broad sampling of clinically-relevant phenotypic information, it may enable multiple genomic investigations upon a single set of genotyped individuals. This chapter reviews several examples of phenotype extraction and their application to genetic research, demonstrating a viable future for genomic discovery using EHR-linked data.

**What to Learn in This Chapter**

- Describe the types of information available in Electronic Health Records (EHRs), and the relative sensitivity and positive predictive value of each
- Describe the difference between unstructured and structured information in the EHR
- Describe methods for developing accurate phenotype algorithms that integrate structured and unstructured EHR information, and the roles played by billing codes, laboratory values, medication data, and natural language processing
- Describe recent uses of EHR-derived phenotypes to study genome-phenome relationships
- Describe the cost advantages unique to EHR-linked biobanks, and the ability to reuse genetic data for many studies
- Understand the role of EHRs to enable phenome-wide association studies of genetic variants

This article is part of the “Translational Bioinformatics” collection for *PLOS Computational Biology*.

## 1. Introduction and Motivation

Typical genetic research studies have used purpose-built cohorts or observational studies for genetic research. As of 2012, more than 1000 genome-wide association analyses have been performed, not to mention a vast quantity of candidate gene studies [1]. Many of these studies have investigated multiple disease and phenotypic traits within a single patient cohort, such as the Wellcome Trust [2] and Framingham research cohorts [3]–[5]. Typically, patient questionnaires and/or research staff are used to ascertain phenotypic traits for a patient. While these study designs may offer high validity and repeatability in their assessment of a given trait, these models are typically very costly and often represent only a cross-section of time. In addition, rare diseases may take a significant time to accrue in these datasets.

Another model that is gaining acceptance is genetic discovery based solely or partially from phenotype information derived solely from the electronic health record (EHR) [6]. In these models, a hospital collects DNA for research, and maintains a linkage between the DNA sample and the EHR data for that patient. The primary source of phenotypic information, therefore, is the EHR. Depending on the design of the biobank model, some EHR-linked biobanks have the ability to supplement EHR-accrued data with purpose-collected research data.

The EHR model for genetic research offers several key advantages, but also faces prominent challenges to successful implementation. A primary advantage is cost. EHRs contain a longitudinal record of robust clinical data that is produced as a byproduct of routine clinical care. Thus, it is a rich, real-world dataset that requires little additional funding to obtain. Both study designs share costs for obtaining and storing DNA.

Another advantage of EHR-linked DNA databanks is the potential to reuse genetic information to investigate a broad range of additional phenotypes beyond the original study. This is particularly true for dense genetic data such as generated through genome-wide association studies or large-scale sequencing data. For instance, a patient may be genotyped once as part of a study on diabetes, and then later participate in another analysis for cardiovascular disease.

Major efforts in EHR DNA biobanking are underway at a number of institutions. One of the major driving forces has been the National Human Genome Research Institute (NHGRI)-sponsored Electronic Medical Records and Genomics (eMERGE) network [7], which began in 2007 and, as of 2012, consists of nine sites that are performing genome-wide association studies using phenotypic data derived from EHR. The National Institutes of Health (NIH)-sponsored Pharmacogenomics Research Network (PGRN) also include sites performing genetic research using EHR data as their source of phenotypic data. Another example is the Kaiser Permanente Research Program on Genes, Environment and Health, which has genotyped 100,000 members with linked EHR data [8].

## 2. Classes of Data Available in EHRs

EHRs are designed primarily to support clinical care, billing, and, increasingly, other functions such as quality improvement initiatives aimed at improving the health of a population. Thus, the types of data and their methods of storing this data are optimized to support these missions. The primary types of information available from EHRs are: billing data, laboratory results and vital signs, provider documentation, documentation from reports and tests, and medication records. Billing data and many laboratory results are available in most systems as structured “name-value pair” data. Clinical documentation, many test results (such as echocardiograms and radiology testing), and medication records are often found in narrative or semi-narrative text formats. Researchers creating “electronic phenotype algorithms” (discussed in Section 6.2) typically utilize multiple types of informatics (e.g., billing codes, laboratory results, medication data, and/or NLP) to achieve high accuracy when identifying cases and controls from the EHR.

Table 1 summarizes the types of data available in the EHR and their strengths and weaknesses.

**Table 1:** Strengths and weakness of data classes within EHRs.

	ICD codes	CPT codes	Laboratory Data	Medication records	Clinical Documentation
**Availability in EHR systems**	Near-universal	Near-universal	Near-universal	Variable	Variable
**Recall**	Medium	Poor	Medium	Inpatient: HighOutpatient: Variable	Medium
**Precision**	Medium	High	High	Inpatient: HighOutpatient: Variable	Medium-High
**Fragmentation effect**	Medium	High	Medium-High	Medium	Low-Medium
**Query method**	Structured	Structured	Mostly structured	Structured, text queries, and NLP	NLP, text queries, and rarely structured
**Strengths**	-Easy to query-Serves as a good first pass of disease status	-Easy to query-High precision	-Value depends on test-High data validity	Can have high validity	Best record of what providers thought
**Weaknesses**	-Disease codes often used for screening when disease not actually present-Accuracy hindered by billing realities and clinic workflow	-Most susceptible to missing data errors (e.g., performed at another hospital)-Procedure receipt influenced by patient and payer factors external to disease process	-May need to aggregate different variations of the same data elements-Normal ranges and units may change over time	-Often need to interface inpatient and outpatient records-Medication records from outside providers not present-Medications prescribed not necessary taken	-Difficult to process automatically-Interpretation accuracy depends on assessment method-May suffer from significant “cut and paste”-Not universally available in EHRs-May be self-contradictory
**Summary**	Essential first element for electronic phenotyping	Helpful addition if relevant	Helpful addition if relevant	Useful for confirmation and a marker of severity	Useful for confirming common diagnoses or for finding rare ones

### 2.1 Billing Data

Billing data typically consists of codes derived from the International Classification of Diseases (ICD) and Current Procedural Terminology (CPT). ICD is a hierarchical terminology of diseases, signs, symptoms, and procedure codes maintained by the World Health Organization (WHO). While the majority of the world uses ICD version 10, the United States (as of 2012) uses ICD version 9-CM; the current Center for Medicare and Medicaid Services guidelines mandate a transition to ICD-10-CM in the United States by October 1, 2014. Because of their widespread use as required components for billing, and due to their ubiquity within EHR systems, billing codes are frequently used for research purposes [9]–[14]. Prior research has demonstrated that such administrative data can have poor sensitivity and specificity [15], [16]. Despite this, they remain an important part of more complex phenotype algorithms that achieve high performance [17]–[19].

CPT codes are created and maintained by the American Medical Association. They serve as the chief coding system providers use to bill for clinical services. Typically, CPTs are paired with ICD codes, the latter providing the reason (e.g., a disease or symptom) for a clinical encounter or procedure. This satisfies the requirements of insurers, who require certain allowable diagnoses and symptoms to pay for a given procedure. For example, insurance companies will pay for a brain magnetic resonance imaging (MRI) scan that is ordered for a number of complaints (such as known cancers or symptoms such as headache), but not for unrelated symptoms such as chest pain.

**Figure 1:**
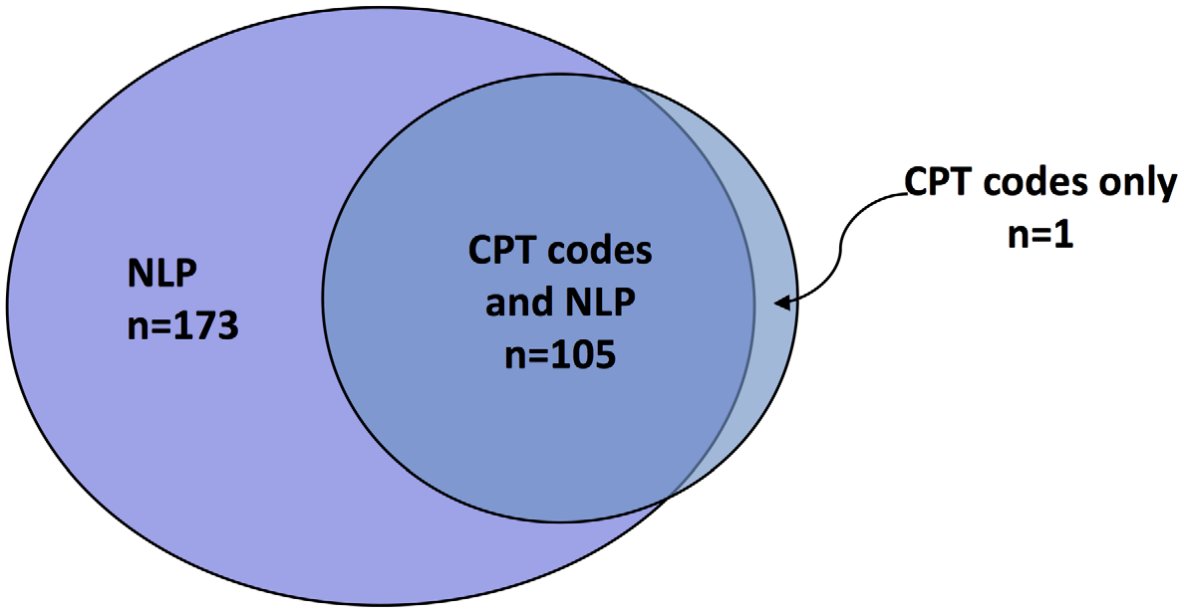
Comparison of natural language processing (NLP) and CPT codes to detect completed colonoscopies in 200 patients. In this study, more completed colonoscopies were found via NLP than with billing codes alone, and only one colonoscopy was found with billing codes that was not found with NLP. NLP examples were reviewed for accuracy.

Within the context of establishing a particular diagnosis from EHR data, CPT codes tend to have high specificity but low sensitivity, while ICD9 codes have comparatively lower specificity but higher sensitivity. For instance, to establish the diagnosis of coronary artery disease, one could look for a CPT code for “coronary artery bypass surgery” or “percutaneous coronary angioplasty” disease, or for one of several ICD9 codes. If the CPT code is present, there is a high probability that the patient has corresponding diagnosis of coronary disease. However, many patients without these CPT codes also have coronary disease, but either have not received these interventions or received them at a different hospital. In contrast, a clinician may bill an ICD9 code for coronary disease based on clinical suspicion without a firm diagnosis. Figure 1 shows the results of a study that compared the use of natural language processing (NLP) and CPT codes to detect patients who have received colorectal cancer screening, via a colonoscopy within the last ten years, at one institution. In this study, only 61% (106 out of 174 total) of the documented completed colonoscopies were found via CPT codes [20]. The most common cause of false negatives was a colonoscopy completed at another hospital. CPT codes, however, had a very high precision (i.e., positive predictive value; see Box 1), with only one false positive.

**Box 1. Metrics Commonly Used to Evaluate Phenotype Selection Algorithms**

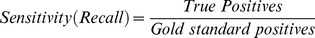

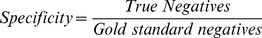

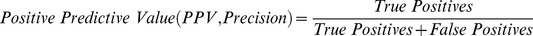

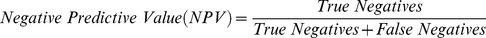

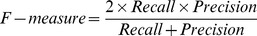

### 2.2 Laboratory and Vital Signs

Laboratory data and vital signs form a longitudinal record of mostly structured data in the medical record. In addition to being stored as name-value pair data, these fields and values can be encoded using standard terminologies. The most common controlled vocabulary used to represent laboratory tests and vital signs is the Logical Observation Identifiers Names and Codes (LOINC®), which is a Consolidated Health Informatics standard for representation of laboratory and test names and is part of Health Language 7 (HL7) [21], [22]. Despite the growing use of LOINC, many (perhaps most) hospital lab systems still use local dictionaries to encode laboratory results internally. Hospital laboratory systems or testing companies may change over time, resulting in different internal codes for the same test result. Thus, care is needed to implement selection logic based on laboratory results. Indeed, a 2009–2010 data standardization effort at Vanderbilt University Medical Center found that the concept of “weight” and “height” each had more than five internal representations. Weights and heights were also recorded by different systems using different field names and stored internally with different units (e.g., kilograms, grams, and pounds for weight; centimeters, meters, inches, and feet for height).

Structured laboratory results are often a very important component of phenotype algorithms, and can represent targets for genomic investigation [3], [4], [23]. An algorithm to identify type 2 diabetes (T2D) cases and controls, for instance, used laboratory values (e.g., hemoglobin A1c and glucose values) combined with billing codes and medication mentions [17]. Similarly, an algorithm to determine genomic determinants of normal cardiac conduction required normal electrolyte (potassium, calcium, and magnesium) values [16]. In these settings, investigation of the determinants of the values requires careful selection of the value to be investigated. For instance, an analysis of determinants of uric acid or red blood cell indices would exclude patients treated with certain antineoplastic agents (which can increase uric acid or suppression of erythrocyte production), and, similarly, an analysis of white blood cell indices also excludes patients with active infections and certain medications at the time of the laboratory measurement.

### 2.3 Provider Documentation

Clinical documentation represents perhaps the richest and most diverse source of phenotype information. Provider documentation is required for nearly all billing of tests and clinical visits, and is frequently found in EHR systems. To be useful for phenotyping efforts, clinical documentation must be in the form of electronically-available text that can be used for subsequent manual review, text searches, or NLP. They can be created via computer-based documentation (CBD) systems or dictated and transcribed. The most common form of computable text is in unstructured narrative text documents, although a number of developers have also created structured documentation tools [24]. Narrative text documents can be processed by text queries or by NLP systems, as discussed in the following section.

**Figure 2:**
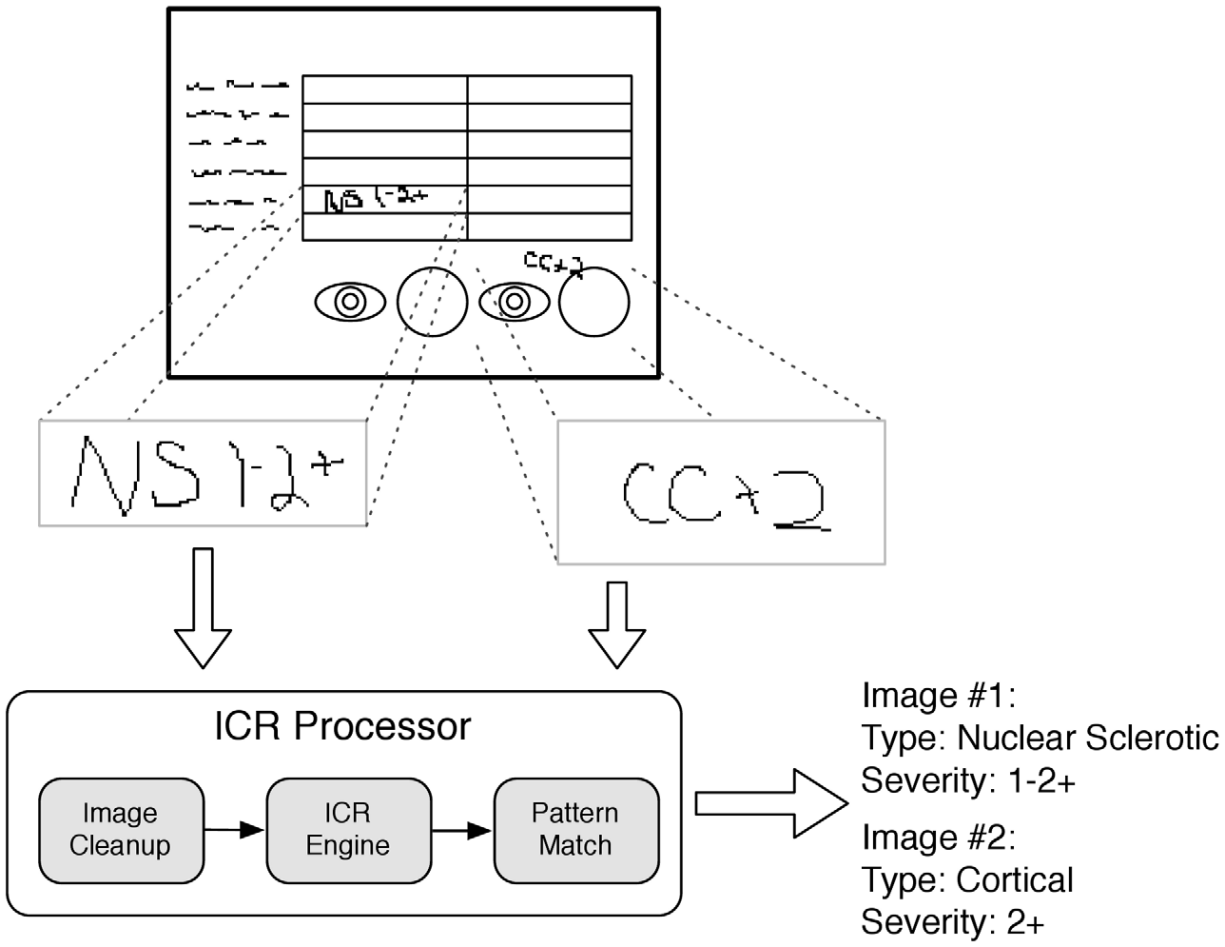
Use of Intelligent Character Recognition to codify handwriting. Figure courtesy of Luke Rasmussen, Northwestern University.

For some phenotypes, crucial documents may only be available as hand-written documents, and thus not amenable to text searching or NLP. Unavailability may result from clinics that are slow adopters, have very high patient volumes, or have specific workflows not well accommodated by the EHR system [25]. However, these hand-written documents may be available electronically as scanned copies. Recent efforts have shown that intelligent character recognition (ICR) software may be useful for processing scanned documents containing hand-written fields (Figure 2) [26], [27]. This task can be challenging, however, and works best when the providers are completing pre-formatted forms.

### 2.4 Documentation from Reports and Tests

Provider-generated reports and test results include radiology and pathology reports and some procedure results such as echocardiograms. They are often in the form of narrative text results. Many of these contain a mixture of structured and unstructured results. Examples include an electrocardiogram report, which typically has structured interval durations and may contain a structured field indicating whether the test was abnormal or not. However, most electrocardiogram (ECG) reports also contain a narrative text “impression” representing the cardiologist's interpretation of the result (e.g., “consider anterolateral myocardial ischemia” or “Since last ECG, patient has developed atrial fibrillation”) [28]. For ECGs, the structured content (e.g., the intervals measured on the ECG) are generated using automated algorithms and have varying accuracy [29].

### 2.5 Medication Records

Medication records serve an important role in accurate phenotype characterization. They can be used to increase the precision of case identification, and to help ensure that patients believed to be controls do not actually have the disease. Medications received by a patient serve as confirmation that the treating physician believed the disease was present to a sufficient degree that they prescribed a treating medication. It is particularly helpful to find presence or absence of medications highly specific or sensitive for the disease. For instance, a patient with diabetes will receive either oral or injectable hypoglycemic agents; these medications are both highly sensitive and specific for treating diabetes, and can also be used to help differentiate type I diabetes (treated almost exclusively with insulin) from T2D (which is typically a disease of insulin resistance and thus can be treated with a combination of oral and injectable hypoglycemic agents).

Medication records can be in varying forms within an electronic record. With the increased use of computerized provider order entry (CPOE) systems to manage hospital stays, inpatient medication records are often available in highly structured records that may be mapped to controlled vocabularies. In addition, many hospital systems are installing automated bar-code medication administration records by which hospital staff record each individual drug administration for each patient [30]. With this information, accurate drug exposures and their times can be constructed for each inpatient. Even without electronic medication administration records (such as bar-code systems), research has shown that CPOE-ordered medications are given with fairly high reliability [31].

Outpatient medication records are often recorded via narrative text entries within clinical documentation, patient problem lists, or communications with patients through telephone calls or patient portals. Many EHR systems have incorporated outpatient prescribing systems, which create structured medical records during generation of new prescriptions and refills. However, within many EHR systems, electronic prescribing tools are optional, not yet widely adopted, or have only been used within recent history. Thus, accurate construction of a patient's medication exposure history often requires NLP techniques. For specific algorithms, focused free-text searching for a set of medications can be efficient and effective [17]. This approach requires the researcher to generate the list of brand names, generics, combination medications, and abbreviations that would be used, but has the advantage that it can be easily accomplished using relational database queries. The downside is that this approach requires re-engineering for each medication or set of medications to be searched, and does not allow for the retrieval of other medication data, such as dose, frequency, and duration. A more general-purpose approach can be achieved with NLP, which is discussed in greater detail in Section 3 below.

## 3. Natural Language Processing to Support Clinical Knowledge Extraction

Although many documentation tools include structured and semi-structured elements, the vast majority of computer based documentation (CBD) remains in “natural language” narrative formats [24]. Thus, to be useful for data mining, narrative data must be processed through use of text-searching (e.g., keyword searching) or NLP systems. Keyword searching can effectively identify rare physical exam findings in text [32], and extension to use of regular expression pattern matching has been used to extract blood pressure readings [33]. NLP computer algorithms scan and parse unstructured “free-text” documents, applying syntactic and semantic rules to extract structured representations of the information content, such as concepts recognized from a controlled terminology [34]–[37]. Early NLP efforts to extract medical concepts from clinical text documents focused on coding in the Systematic Nomenclature of Pathology or the ICD for financial and billing purposes [38], while more recent efforts often use complete versions of the Unified Medical Language System (UMLS) [39]–[41], SNOMED-CT [16], and/or domain-specific vocabularies such as RxNorm for medication extraction [42]. NLP systems utilize varying approaches to “understanding text,” including rule-based and statistical approaches using syntactic and/or semantic information. Natural language processors can achieve classification rates similar to those of manual reviewers, and can be superior to keyword searches. A number of researchers have demonstrated the effectiveness of NLP for large-scale text-processing tasks. Melton and Hripcsak used MedLEE to recognize instances of adverse events in hospital discharge summaries [43]. Friedman and colleagues evaluated NLP for pharmacovigilance to discover adverse drug events from clinical records by using statistical methods that associate extracted UMLS disease concepts with extracted medication names [40]. These studies show the potential for NLP to aid in specific phenotype recognition.

Using either NLP systems or keyword searching, the primary task in identifying a particular phenotype is to filter out concepts (or keywords) within a corpus of documents that indicate statements other than the patient having the disease. Researchers may desire to specify particular document types (e.g., documents within a given domain, problem lists, etc.) or particular types of visits or specialists (e.g., requiring a visit with an ophthalmologist). Some common NLP tasks needed in phenotype classification include identifying family medical history context and negated terms (e.g., “no cardiac disease”), and removing drug allergies when searching for patients taking a certain medication. Recognition of sections within documents can be handled using structured section labels, specialized NLP systems such as SecTag [44], or more general-purpose NLP systems such as MedLEE [45] or HITEX [46]. A number of solutions have been proposed for negation detection; among the more widespread are adaptations of the NegEx algorithm developed by Chapman et al., which uses a series of negation phrases and boundary words to identify negated text [47]. NegEx or similar algorithms can be used as a standalone system or be integrated within a number of general-purpose NLP systems including MedLEE [48], the KnowledgeMap concept identifier [49], cTAKES [50], and the National Library of Medicine's MetaMap [51].

Medication information extraction is an important area for clinical applications that benefits from specialized NLP tools. Most general-purpose NLP systems will recognize medications by the medication ingredient mentioned in the text but may not identify the relevant medication metadata such as dose, frequency, and route. In addition, a general purpose NLP system using as its vocabulary the UMLS will likely recognize “atenolol” and “Tenormin” (a United States brand name for atenolol) as two different concepts, since each is represented by separate concepts in the UMLS. Medication-specific NLP systems focus on extracting such metadata for a medication. Sirohl and Peissig applied a commercial medication NLP system to derived structured medication information [52], which was later linked to laboratory data and used to explore the pharmacodynamics of statin efficacy (a cholesterol-lowering medication) [53]. Xu et al. developed a similar system at Vanderbilt called MedEx, which had recall and precision ≥0.90 for discharge summaries and clinic notes on Vanderbilt clinical documents [42]. Additionally, the 2009 i2b2 NLP challenge focused on medication extraction using de-identified discharge summaries from Partners Healthcare, and 20 teams competed to identify medications and their signatures. The best systems achieved F-measures ≥0.80 [54]. Much work remains to be done in this area, as extraction of both medication names and associated signature information can be challenging when considering the full breadth of clinical documentation formats available, including provider-staff and provider-patient communications, which often contain less formal and misspelled representations of prescribed medications.

For more information on NLP methods and applications, please see the article on text mining elsewhere in this collection (submitted).

## 4. EHR-Associated Biobanks: Enabling EHR-Based Genomic Science

DNA biobanks associated with EHR systems can be composed of either “all comers” or a focused collection, and pursue either a conventional consented “opt-in” or an “opt-out” approach. Currently, the majority of DNA biobanks have an opt-in approach that selects patients for particular research studies. Two population-based models in the eMERGE network are the Personalized Medicine Research Population (PMRP) project of the Marshfield Clinic (Marshfield, WI) [55] and Northwestern University's NUgene project (Chicago, IL). The PMRP project selected 20,000 individuals who receive care in the geographic region of the Marshfield Clinic. These patients have been consented, surveyed, and have given permission to the investigators for recontact in the future if additional information is needed. The NUgene project, which has enrolled nearly 10,000 people through 2012, uses a similar approach, obtaining patients' consent during outpatient clinic visits [56]. Another example of an EHR-associated biobank is the Kaiser-Permanente biobank, which has genotyped 100,000 individuals [57].

The alternative “opt-out” approach is evidenced by Vanderbilt University's BioVU, which associates DNA with de-identified EHR data [58]. In this model, patients have the opportunity to “opt out” of the DNA biobank by checking a box on the standard “Consent to Treatment” form signed as part of routine clinical care. A majority of patients (>90%) do not check this box, indicating assent to the use of their DNA in the biobank [58]. If the patient does not opt-out, blood that is scheduled to be discarded after routine laboratory testing is instead sent for DNA extraction, which is stored for potential future use. To ensure that no one knows with certainty if a subject's DNA is in BioVU, an additional small percentage of patients are randomly excluded.

The BioVU model requires that the DNA and associated EHR data be de-identified in order to assure that the model complies with the policies of non-human subjects research. The full-text of the EHR undergoes a process of de-identification with software programs that remove Health Insurance Portability and Accountability Act (HIPAA) identifiers from all clinical documentation in the medical record. At the time of this writing, text de-identification for BioVU is performed using the commercial product DE-ID [59] with additional pre- and post-processing steps. However, a number of other clinical text de-identification software packages have been studied, some of which are open source [60], [61]. Multiple reviews by both the local institutional review board and the federal Office for Human Research Protections have affirmed this status as nonhuman subjects research according to 45 CFR 46 [58]. Nonetheless, all research conducted within BioVU and the associated de-identified EHR (called the “Synthetic Derivative”) is overseen by the local Institutional Review Board. An opt-out model similar to BioVU is used by Partners Healthcare for the Crimson biobank, which can accrue patients who meet specific phenotype criteria as they have routine blood draws.

An advantage of the opt-out approach is rapid sample accrual. BioVU began collecting DNA samples in 2007, adding about 500 new samples weekly, and has over 150,000 subjects as of September 2012. Since it enrolls subjects prospectively, investigation of rare phenotypes may be possible with such systems. The major disadvantage of the opt-out approach is that it precludes recontact of the patients since their identity has been removed. However, the Synthetic Derivative is continually updated as new information is added to the EHR, such that the amount of phenotypic information for included patients grows over time.

## 5. Race and Ethnicity in EHR-Derived Biobanks

Given that much genetic information varies greatly within ancestral populations, accurate knowledge of genetic ancestry information is essential to allow for proper genetic study design and control of population stratification. Without it, one can see numerous spurious genetic associations due solely to race/ethnicity [62]. Single nucleotide polymorphisms (SNPs) common in one population may be rare in another. In large-scale GWA analyses, one can tolerate less accurate knowledge of ancestry *a priori*, since the large amount of genetic data allows one to calculate the genetic ancestry of the subject using catalogs of SNPs known to vary between races. Alternatively, one can also adjust for genetic ancestry using tools such as EIGENSTRAT [63]. However, in smaller candidate gene studies, it is important to know the ancestry beforehand.

Self-reported race/ethnicity data is often used in genetic studies. In contrast race/ethnicity as recorded within an EHR may be entered through a variety of sources. Most commonly, administrative staff record race/ethnicity via structured data collection tools in the EHR. Often, this field can be ignored (left as “unknown”), especially in busy clinical environments, such as emergency departments. “Unknown” percentages of patients can range between 9% and 23% of subjects [17], [18]. Among those patients for whom data is entered, a study of genetic ancestry informative markers correlated well with EHR-reported race/ethnicities [64]. In addition, a study within the Veterans Administration (VA) hospital system noted that over 95% of all EHR-derived race/ethnicity agreed with self-reported race/ethnicity using nearly one million records [65]. Thus, despite concerns over EHR-derived ancestral information, such information, when present, appears similar to self-report ancestry information.

## 6. Phenotype-Driven Discovery in EHRs

### 6.1 Measure of Phenotype Selection Logic Performance

The evaluation of phenotype selection logic can use metrics similar to information retrieval tasks. Common metrics are sensitivity (or recall), specificity, positive predictive value (PPV, also known as precision), and negative predictive value (see Box 1). If a population is assessed for case and control status, then another useful metric is comparing the receiver operator characteristic (ROC) curves. ROC curves graph the sensitivity vs. false positive rate (or, 1-specificity) given a continuous measure of the outcome of the algorithm. By calculating the area under the ROC curve (AUC), one has a single measure of the overall performance of an algorithm that can be used to compare two algorithms or selection logics. Since the scale of the graph is 0 to 1 on both axes, the performance of a perfect algorithm is 1, and random chance is 0.5.

### 6.2 Creation of Phenotype Selection Logic

Initial work in phenotype detection has often focused on a single modality of EHR data. A number of studies have used billing data, some comparing directly to other genres of data, such as NLP. Li et al. compared the results of ICD-9 encoded diagnoses and NLP-processed discharge summaries for clinical trial eligibility queries, finding that use of NLP provided more valuable data sources for clinical trial pre-screening than ICD-9 codes [15]. Savova et al. has used cTAKES to discover peripheral arterial disease cases by looking for particular key words in radiology reports, and then aggregating the individual instances using “AND-OR-NOT” Boolean logic to classify cases into four categories: positive, negative, probable, and unknown [66].

**Figure 3:**
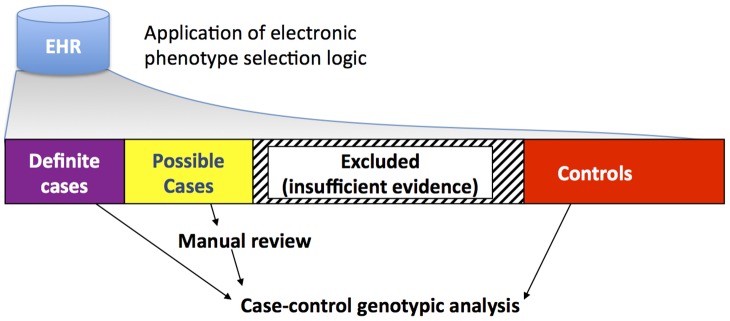
General figure for identifying cases and controls using EHR data. Application of electronic selection algorithms lead to division of a population of patients into four groups, the largest of which comprises patients who were excluded because they lack sufficient evidence to be either a case or control patient. Definite cases and controls cross some predefined threshold of positive predictive value (e.g., PPV≥95%), and thus do not require manual review. For very rare phenotypes or complicated case definitions, the category of “possible” cases may need to be reviewed manually to increase the sample size.

Phenotype algorithms can be created multiple ways, depending of the rarity of the phenotype, the capabilities of the EHR system, and the desired sample size of the study. Generally, phenotype selection logics (algorithms) are composed of one or more of four elements: billing code data, other structured (coded) data such as laboratory values and demographic data, medication information, and NLP-derived data. Structured data can be retrieved effectively from most EHR systems. These data can be combined through simple Boolean logic [17] or through machine learning methods such as logistic regression [18], to achieve a predefined specificity or positive predictive value. A drawback to the use of machine learning data (such as logistic regression models) is that it may not be as portable to other EHR systems as more simple Boolean logic, depending on how the models are constructed. The application of many phenotype selection logics can be thought of partitioning individuals into four buckets – definite cases (with sufficiently high PPV), possible cases (which can be manually reviewed if needed), controls (which do not have the disease with acceptable PPV), and individuals excluded from the analysis due to either potentially overlapping diagnoses or insufficient evidence (Figure 3).

For many algorithms, sensitivity (or recall) is not necessarily evaluated, assuming there are an adequate number of cases. A possible concern in not evaluating recall (sensitivity) of a phenotype algorithm is that there may be a systematic bias in how patients were selected. For example, consider a hypothetical algorithm to find patients with T2D whose logic was to select all patients that had at least one billing code for T2D and also required that cases receive an oral hypoglycemic medication. This algorithm may be highly specific for finding patients with T2D (instead of type 1 diabetes), but would miss those patients who had progressed in disease severity such that oral hypoglycemic agents no longer worked and who now require insulin treatment. Thus, this phenotype algorithm could miss the more severe cases of T2D. However, for a practical application, such assessments of recall can be challenging given large samples sizes of rare diseases. Certain assumptions (e.g., that a patient should have at least one billing code for the disease) are reasonable and likely do not lead to significant bias.

For other algorithms, the temporal relationships of certain elements are very important. Consider an algorithm to determine whether a certain combination of medication adversely impacted a given lab, such as kidney function or glucose [67]. Such an algorithm would need to take into account the temporal sequence and time between the particular medications and laboratory tests. For example, glucose changes within minutes to hours of a single administration of insulin, but the development of glaucoma from corticosteroids (a known side effect) would not be expected to happen acutely following a single dose.

For very rare diseases or findings, one may desire to find every case, and thus the logic may simply be a union of keyword text queries and billing codes followed by manual review of all returned cases. Examples include the rare physical exam finding hippus (exaggerated pupillary oscillations occurring in the setting of altered mental status) [32], or potential drug adverse events (e.g., Stevens-Johnson syndrome), which are often very rare but severe.

Since EHRs represent longitudinal records of patient care, they are biased to recording those events that are recorded as part of medical care. Thus, they are particularly useful for investigating disease-based phenotypes, but potentially less efficacious for investigating non-disease phenotypes such as hair or eye color, left vs. right handedness, cognitive attributes, biochemical measures (beyond routine labs), etc. On the other hand, they may be particularly useful for analyzing disease progression over time.

## 7. Examples of Genetic Discovery Using EHRs

The growth of “EHR-driven genomic research” (EDGR) – that is, genomic research proceeding primarily from EHR data linked to DNA samples – is a recent phenomenon [6]. Preceding these most recent research initiatives, other studies laid the groundwork for use of EHR data to study genetic phenomena. Rzhetsky et al. used billing codes from the EHRs of 1.5 million patients to analyze disease co-occurrence in 161 conditions as a proxy for possible genetic overlap [68]. Chen et al. compared laboratory measurements and age with gene expression data to identify rates of change that correlated with genes known to be involved in aging [69]. A study at Geisinger Clinic evaluated SNPs in the 9p21 region that are known to be associated to cardiovascular disease and early myocardial infarction [70]. They found these SNPs were associated with heart disease and T2D using EHR-derived data. Several specific examples of EDGR are detailed below.

### 7.1 Replicating Known Genetic Associations for Five Diseases

An early replication study of known genetic associations with five diseases with known genetic associations was performed in BioVU. The study was designed to test the hypothesis that an EHR-linked DNA biobank could be used for genetic association analyses. The goal was to use only EHR data for phenotype information. The first 10,000 samples accrued in BioVU were genotyped at 21 SNPs that are known to be associated with these five diseases (atrial fibrillation, Crohn's disease, multiple sclerosis, rheumatoid arthritis, and T2D). Reported odds ratios were 1.14–2.36 in at least two previous studies prior to the analysis. Automated phenotype identification algorithms were developed using NLP techniques (to identify key findings, medication names, and family history), billing code queries, and structured data elements (such as laboratory results) to identify cases (n = 70–698) and controls (n = 808–3818). Final algorithms achieved PPV of ≥97% for cases and 100% for controls on randomly selected cases and controls (Table 2) [17]. For each of the target diseases, the phenotype algorithms were developed iteratively, with a proposed selection logic applied to a set of EHR subjects, and random cases and controls evaluated for accuracy. The results of these reviews were used to refine the algorithms, which were then redeployed and reevaluated on a unique set of randomly selected records to provide final PPVs.

**Table 2:** Methods of finding cases and controls for genetic analysis of five common diseases.

Disease	Methods	Cases	Controls	Case PPV	Control PPV
Atrial fibrillation	NLP of ECG impressionsICD9 codesCPT codes	168	1695	98%	100%
Crohn's Disease	ICD9 codesMedications (text)	116	2643	100%	100%
Type 2 Diabetes	ICD9 codesMedications (text)Text searches (controls)	570	764	100%	100%
Multiple Sclerosis	ICD9 codes or text diagnosis	66	1857	87%*	100%
Rheumatoid Arthritis	ICD9 codesMedications (text)Text searches (exclusions)	170	701	97%	100%

* Given the small number of multiple sclerosis cases, all possible cases were manually validated to ensure high recall.

Used alone, ICD9 codes had PPVs of 56–89% compared to a gold standard represented by the final algorithm. Errors were due to coding errors (e.g., typos), misdiagnoses from non-specialists (e.g., a non-specialist diagnosed a patient as having rheumatoid arthritis followed by a rheumatologist who revised the diagnosis to psoriatic arthritis), and indeterminate diagnoses that later evolved into well-defined ones (e.g., a patient thought to have Crohn's disease was later determined to have ulcerative colitis, another type of inflammatory bowel disease). Each of the 21 tests of association yielded point estimates in the expected direction, and eight of the known associations achieved statistical significance [17].

### 7.2 Demonstrating Multiethnic Associations with Rheumatoid Arthritis

Using a logistic regression algorithm operating on billing data, NLP-derived features, medication records, and laboratory data, Liao et al. developed an algorithm to accurately identify rheumatoid arthritis patients [18]. Kurreeman et al. used this algorithm on EHR data to identify a population of 1,515 cases and 1,480 matched controls [71]. These researchers genotyped 29 SNPs that had been associated with RA in at least one prior study. Sixteen of these SNPs achieved statistical significance, and 26/29 had odds ratios in the same direction and with similar effect sizes. The authors also demonstrated that these portions of these risk alleles were associated with rheumatoid arthritis in East Asian, African, and Hispanic American populations.

### 7.3 eMERGE Network

The eMERGE network is composed of nine institutions as of 2012 (http://gwas.org; Table 3). Each site has a DNA biobank linked to robust, longitudinal EHR data. The initial goal of the eMERGE network was to investigate the feasibility of genome-wide association studies using EHR data as the primary source for phenotypic information. Each of these sites initially set out to investigate one or two primary phenotypes (Table 3). Network sites have currently created and evaluated electronic phenotype algorithms for 14 different primary and secondary phenotypes, with nearly 30 more planned. After defining phenotype algorithms, each site then performed genome-wide genotyping at one of two NIH-supported genotyping centers.

**Table 3:** eMERGE network participants.

Institution	Biorepository Overview	Model	Size	EHR Summary	Phenotyping Methods
**Group Health** 1(Seattle, WA)	**GHC Biobank**Alzheimer's DiseasePatient Registry and Adult Changes in Thought Study	Disease specificCohort	4000	Comprehensive vendor-based EHR since 2004	Structured data extraction, NLP
**Marshfield Clinic Research Foundation** 1(Marshfield, WI)	**Personalized Medicine Research Project**Marshfield Clinic, an integrated regional health system	Population based	20,000	Comprehensive internally developed EHR since 1985	Structured data extraction, NLP,Intelligent Character Recognition
**Mayo Clinic** 1(Rochester, MN)	**Disease cohort**Derived from vascular laboratory & exercise stress testing labs	Disease specificCohorts	16,500	Comprehensive internally developed EHR since 1995	Structured data extraction, NLP
**Northwestern University** 1(Chicago, IL)	**NUgene Project**Northwestern affiliated hospitals and outpatient clinics	Population based	>10,000	Comprehensive vendor based Inpatient and Outpatient (different systems) EHR since 2000	Structured data extraction, text searches, NLP
**Vanderbilt University** 1(Nashville, TN)	**BioVU**Primarily drawn from outpatient routine laboratory samples	Population based	150,000	Comprehensive internally developed EHR since 2000	Structured data extraction, NLP
**Geisinger Health System** 2 (Pennsylvania)	**MyCode**Enrollment of health plan participants	Population based	>30,000	Comprehensive vendor-based EHR	Structured data extraction, NLP
**Mount Sinai Medical Center** 2 (New York, NY)	**Institute for Personalized Medicine Biobank**Outpatient enrollment	Population based	>30,000	Comprehensive vendor-based EHR since 2004	Structured data extraction, NLP
**Cincinnati Children's Hospital** 3(Cincinnati, OH)	General and disease cohorts.	Population based	>3,000	Comprehensive vendor-based EHR	Structured data extraction, NLP
**Children's Hospital of Philadelphia** 3(Philadelphia, PA)	General and disease cohorts.	Population based	>100,000	Comprehensive vendor-based EHR	Structured data extraction, NLP
**Boston Children's** 3(Boston MA)	**Crimson**On-demand, de-identified phenotype-driven collection	Disease based	Virtual	Comprehensive internally developed EHR	Structured data extraction, NLP

Sizes represent approximate sizes as of 2012; many sites are still actively recruiting. NLP = Natural Language Processing. Sites joined with ^1^eMERGE-I in 2007, ^2^eMERGE-II in 2011, or as ^3^pediatric sites in 2012.

The primary goals of an algorithm are to perform with high precision (≥95%) and reasonable recall. Algorithms incorporate billing codes, laboratory and vital signs data, test and procedure results, and clinical documentation. NLP is used to both increase recall (find additional cases) and achieve greater precision (via improved specificity). These phenotype algorithms are available for download from PheKB (http://phekb.org).

Initial plans were for each site to analyze their own phenotypes independently. However, the network has realized the benefits of synergy. Central efforts across the network were involved in harmonization of the collective genetic data.

### 7.4 Early Genome-Wide Association Studies from the eMERGE Network

As of 2012, the eMERGE Network has published GWAS on atrioventricular conduction [72], red blood cell [23] and white blood cell [73] traits, primary hypothyroidism [74], and erythrocyte sedimentation rate [75], with others ongoing. The first two studies published by the network were using single-site GWAS studies; latter studies have realized the advantage of pooling data across multiple sites to increase the sample size available for a study. Importantly, several studies in eMERGE have explicitly evaluated the portability of the electronic phenotype algorithms by reviewing algorithms at multiple sites. Evaluation of the hypothyroidism algorithm at the five eMERGE-I sites, for instance, noted an overall weighted PPV of 92.4% and 98.5% for cases and controls, respectively [74]. Similar results have been found with T2D [76], cataracts [27], and rheumatoid arthritis [77] algorithms.

**Figure 4:**
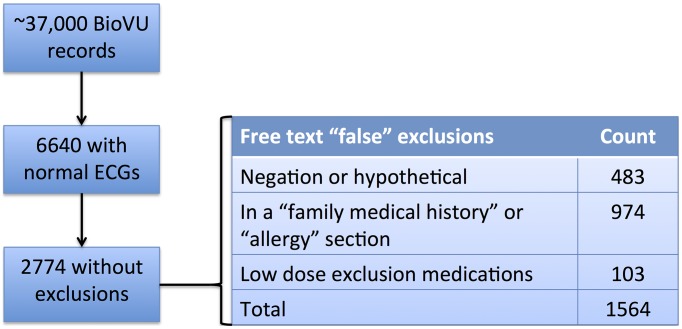
Use of NLP to identify patients without heart disease for a genome-wide analysis of normal cardiac conduction. Using simple text searching, 1564 patients would have been eliminated unnecessarily due to negated terms, family medical history of heart disease, or low dose medication use that would not affect measurements on the electrocardiogram. Use of NLP improves recall of these cases without sacrificing positive predictive value. The final case cohort represented the patients used for GWAS in [71].

As a case study, the GWAS for atrioventricular conduction (as measured by the PR interval on the ECG), conducted entirely within samples drawn from one site, identified variants in *SCN10A*. *SCN10A* is a sodium channel expressed in autonomic nervous system tissue and is now known to be involved in cardiac regulation. The phenotype algorithm identified patients with normal ECGs who did not have evidence of prior heart disease, were not on medications that would interfere with cardiac conduction, and had normal electrolytes. The phenotype algorithm used NLP and billing code queries to search for the presence of prior heart disease and medication use [72]. Of note, the algorithm highlights the importance of using clinical note section tagging and negation to exclude only those patients with heart disease, as opposed to patients whose records contained negated heart disease concepts (e.g., “no myocardial infarction”) or heart disease concepts in related individuals (e.g., “mother died of a heart attack”). Use of NLP improved recall of cases by 129% compared with simple text searching, while maintaining a positive predictive value of 97% (Figure 4) [78], [72].

The study of RBC traits identified four variants associated with RBC traits. One of these, *SLC17A1*, had not been previously identified, and is involved in sodium-phosphate co-transport in the kidney. The latter study of RBC traits utilized patients genotyped at one site as cases and controls for their primary phenotype of peripheral arterial disease (PAD). Thus, this represents an *in silico* GWAS for a new finding that did not require new genotyping, but instead leveraged the available data within the EHR. The eMERGE study of primary hypothyroidism, similarly, identified a novel association with *FOXE1*, a thyroid transcription factor, without any new genotyping by using samples derived from five eMERGE sites.

### 7.5 Phenome-Wide Association Studies (PheWAS)

Typical genetic analyses investigate many genetic loci against a single trait or disease. Such analyses cannot identify pleiotropic associations, and may miss important confounders in an analysis. Another approach, engender by the rich phenotype record included in the EHR, is to simultaneously investigate many phenotypes associated with a given genetic locus. A “phenome-wide association study” (PheWAS) is, in a sense, a “reverse GWAS.” PheWAS investigations require large representative patient populations with definable phenotypic characteristics. Such studies only recently became feasible, facilitated by linkage of DNA biorepositories to EHR systems, which can provide a comprehensive, longitudinal record of disease.

**Figure 5:**
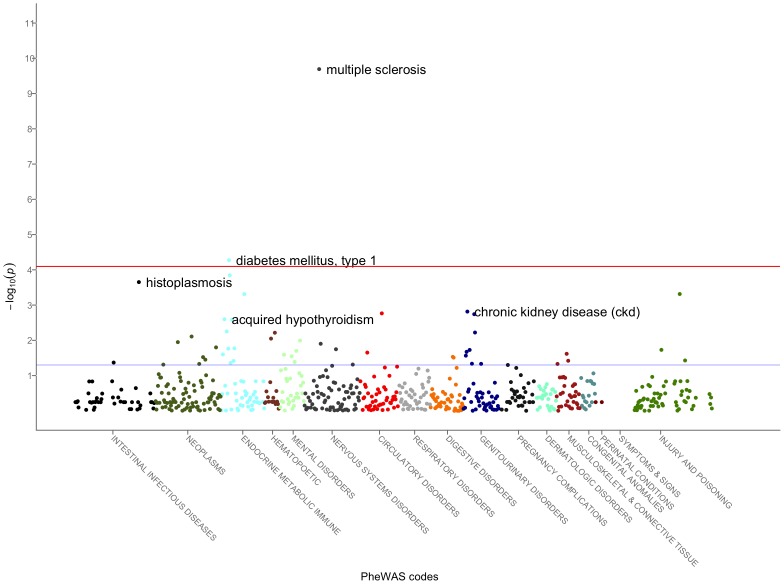
A PheWAS plot for rs3135388 in HLA-DRA. This region has known associations with multiple sclerosis. The red line indicates statistical significance at Bonferroni correction. The blue line represents p<0.05. This plot is generated from updated data from [78] and the updated PheWAS methods as described in [73].

The first PheWAS studies were performed on 6,005 patients genotyped for five SNPs with seven previously known disease associations [79]. This PheWAS used ICD9 codes linked to a code-translation table that mapped ICD9 codes to 776 disease phenotypes. In this study, PheWAS methods replicated four of seven previously known associations with p<0.011. Figure 5 shows one illustrative PheWAS plot of phenotype associations with an *HLA-DRA* SNP known to be associated with multiple sclerosis. Of note, this PheWAS not only demonstrates a strong association between this SNP and multiple sclerosis, but also highlights other possible associations, such as Type 1 diabetes and acquired hypothyroidism. Recent explorations into PheWAS methods using NLP have shown greater efficacy for detecting associations: with the same patients, NLP-based PheWAS replicated six of the seven known associations, generally with more significant p-values [80].

PheWAS methods may be particularly useful for highlighting pleiotropy and clinically associated diseases. For example, an early GWAS for T2D identified, among others, *FTO* loci as an associated variant [81]. A later GWAS demonstrated this risk association was mediated through the effect of *FTO* on increasing body mass index, and thus increasing risk of T2D within those individuals. Such effects may be identified through broad phenome scans made possible through PheWAS.

## 8. Conclusions and Future Directions

EHRs have long been seen as a vehicle to improve healthcare quality, cost, and safety. However, their growing adoption in the United States and elsewhere is demonstrating their capability as a broad tool for research. Enabling tools include enterprise data warehouses and software to process unstructured information, such as de-identification and NLP. When linked to biological data such as DNA or tissue biorepositories, EHRs can become a powerful tool for genomic analysis. One can imagine future repositories also storing intermittent plasma samples to allow for proteomic analyses.

A key advantage of EHR-based genetic studies is that they allow for the collection of phenotype information as a byproduct of routine healthcare. Moreover, this information collection grows over time and is continually refined as new information may confirm or refute a diagnosis for a given individual. Through the course of one's life, a number of information points concerning disease, response to treatment, and laboratory and test data are collected. Aggregation of this information can allow for generation of large sample sizes of patients with certain diseases or medication exposures. Moreover, once a subject receives dense genotyping for one EHR-based study, their genetic data can be reused for many other genotypic studies, allowing for relatively low-cost reuse of the genetic material (once a given phenotype can be found in the EHR).

Three major rate-limiting steps impede utilization of EHR data for genetic analysis. A major challenge is derivation of accurate collections of cases and controls for a given disease of interest, usually achieved through creation and validation of phenotype selection logics. These algorithms take significant time and effort to develop and often require adjustment and a skilled team to deploy at a secondary site. Another challenge is the availability of phenotypic information. Many patients may be observed at a given healthcare facility only for certain types of care (e.g., primary care or a certain subspecialist), leading to fragmented knowledge of a patient's medical history and medication exposures. Future growth of Health Information Exchanges could substantially improve these information gaps. Finally, DNA biobanks require significant institutional investment and ongoing financial, ethical, and logistical support to run effectively. Thus, they are not ubiquitous.

As genomics move beyond discovery into clinical practice, the future of personalized medicine is one in which our genetic information could be “simply a click of the mouse” away [82]. In this future, DNA-enabled EHR systems will assist in more accurate prescribing, risk stratification, and diagnosis. Genomic discovery in EHR systems provides a real-world test bed to validate and discover clinically meaningful genetic effects.

## 9. Exercises

1. Compare and contrast the basic types of data available in an Electronic Health Records (EHR) that are useful for mining genetic data. What are some of the strengths and drawbacks of each type of data?
2. Explain what a phenotype algorithm is and why it is necessary. For example, how can use of natural language processing improve upon use of billing codes alone?
3. Select a clinical disease and design a phenotype algorithm for it.
4. How might a phenotype algorithm be different for a very rare disease (e.g., prion diseases) vs. a more common one (e.g., Type 2 diabetes)? How would a phenotype algorithm be different for a physical exam finding (e.g., hippus or a particular type of heart murmur) vs. a disease?
5. Describe the differences between a DNA biobank linked to an EHR and one collected as part of a non-EHR research cohort. What are the advantages and disadvantages of a de-identified DNA biobank vs. an identified DNA biobank (either linked to an EHR or not).
6. It is often harder to create algorithms to find drug-response phenotypes (such as adverse drug events) than for a chronic disease. Give several reasons why this might be.

Answers to the Exercises can be found in Text S1.

**Further Reading**

- Shortliffe EH, Cimino JJ, editors (2006) Biomedical informatics: computer applications in health care and biomedicine. 3rd edition. Springer. 1064 p. *Chapters of particular relevance: Chapter 2 (“Biomedical data: their acquisition, storage, and use”), Chapter 8 (“Natural language and text processing in biomedicine”), Chapter 12 (“Electronic health record systems”)*
- Hristidis V, editor (2009) Information discovery on electronic health records. 1st edition. Chapman and Hall/CRC. 331 p. *Chapters of particular relevance: Chapter 2 (“Electronic health records”), Chapter 4 (“Data quality and integration issues in electronic health records”), 7 (“Data mining and knowledge discovery on EHRs”).*
- Wilke RA, Xu H, Denny JC, Roden DM, Krauss RM, et al. (2011) The emerging role of electronic medical records in pharmacogenomics. Clin Pharmacol Ther 89: 379–386. doi:10.1038/clpt.2010.260.
- Roden DM, Xu H, Denny JC, Wilke RA (2012) Electronic medical records as a tool in clinical pharmacology: opportunities and challenges. Clin Pharmacol Ther. Available: http://www.ncbi.nlm.nih.gov/pubmed/22534870. Accessed 30 June 2012.
- Kohane IS (2011) Using electronic health records to drive discovery in disease genomics. Nat Rev Genet 12: 417–428. doi:10.1038/nrg2999.

**Glossary**

- **Candidate gene study**: A study of specific genetic loci in which a phenotype-genotype association may exist (e.g., hypothesis-led genotype experiment)
- **Computer-based documentation (CBD)**: Any electronic note or report found within an EHR system. Typically, these can be dictated or typed directly into a “note writer” system (which may leverage “templates”) available within the EHR. Notably, CBD excludes scanned documents.
- **Computerized Provider Order Entry (CPOE)**: A system for allowing a provider (typically a clinician or a nurse practitioner) to enter, electronically, an order for a patient. Typical examples include medication prescribing or test ordering. These systems allow for a precise electronic record of orders given and also can provide decision support to help improve care.
- **Electronic Health Record (EHR)**: Any comprehensive electronic medical record system storing all the data about a patient's encounters with a healthcare system, including medical diagnoses, physician notes, prescribing records. EHRs include CPOE and CBD systems (among others), and allow for easy information retrieval of clinical notes and results.
- **Genome-wide association study (GWAS)**: A broad scale study of a number of points selected along a genome without using a prior hypothesis. Typically, these studies analyze more than >500,000 loci on the genome.
- **Genotype**: The specific DNA sequence at a given location.
- **Natural language processing (NLP)**: Use of algorithms to created structured data from unstructured, narrative text documents. Examples include use of comprehensive NLP software solutions to find biomedical concepts in documents, as well as more focused applications of techniques to find extract features from notes, such as blood pressure readings.
- **Phenome-wide association study (PheWAS)**: A broad scale study of a number phenotypes selected along the genome without regard to a prior hypothesis as what phenotype(s) a given genetic locus may be associated.
- **Phenotype selection logic (or algorithm)**: A series of Boolean rules or machine learning algorithms incorporating such information as billing codes, laboratory values, medication records, and NLP designed to derive a case and control population. from EHR data.
- **Phenotype**: Any observable attribute of an individual.
- **Single nucleotide polymorphism (SNP)**: a single locus on the genome that shows variation in the human population.
- **Structured data**: Data that is already recorded in a system in a structured name-value pair format and can be easily queried via a database.
- **Unified Medical Language System (UMLS)**: A comprehensive metavocabulary maintained by the National Library of Medicine which combines >100 individual standardized vocabularies. The UMLS is composed of the Metathesaurus, the Specialist Lexicon, and the Semantic Network. The largest component of the UMLS is the Metathesaurus, which contains the term strings, concept groupings of terms, and concept interrelationships.
- **Unstructured data**: Data contained in narrative text documents such as the clinical notes generated by physicians and certain types of text reports, such as pathology results or procedures such as echocardiograms.

## Supporting Information

Text S1 Answers to Exercises.
(DOCX)

## References

[1] Hindorff LA, Sethupathy P, Junkins HA, Ramos EM, Mehta JP, et al (2009) Potential etiologic and functional implications of genome-wide association loci for human diseases and traits. Proc Natl Acad Sci USA 106: 9362–9367 doi:10.1073/pnas.0903103106. 19474294 10.1073/pnas.0903103106 PMC2687147

[2] Wellcome Trust Case Control Consortium (2007) Genome-wide association study of 14,000 cases of seven common diseases and 3,000 shared controls. Nature 447: 661–678. 17554300 10.1038/nature05911 PMC2719288

[3] Dehghan A, Köttgen A, Yang Q, Hwang S-J, Kao WL, et al (2008) Association of three genetic loci with uric acid concentration and risk of gout: a genome-wide association study. Lancet 372: 1953–1961 doi:10.1016/S0140-6736(08)61343-4. 18834626 10.1016/S0140-6736(08)61343-4 PMC2803340

[4] Benjamin EJ, Dupuis J, Larson MG, Lunetta KL, Booth SL, et al (2007) Genome-wide association with select biomarker traits in the Framingham Heart Study. BMC Med Genet 8 Suppl 1: S11 doi:10.1186/1471-2350-8-S1-S11. 17903293 10.1186/1471-2350-8-S1-S11 PMC1995615

[5] Kiel DP, Demissie S, Dupuis J, Lunetta KL, Murabito JM, et al (2007) Genome-wide association with bone mass and geometry in the Framingham Heart Study. BMC Med Genet 8 Suppl 1: S14. 17903296 10.1186/1471-2350-8-S1-S14 PMC1995606

[6] Kohane IS (2011) Using electronic health records to drive discovery in disease genomics. Nat Rev Genet 12: 417–428 doi:10.1038/nrg2999. 21587298 10.1038/nrg2999

[7] Manolio TA (2009) Collaborative genome-wide association studies of diverse diseases: programs of the NHGRI's office of population genomics. Pharmacogenomics 10: 235–241. 19207024 10.2217/14622416.10.2.235 PMC2714942

[8] Kaiser Permanente, UCSF Scientists Complete NIH-Funded Genomics Project Involving 100,000 People (n.d.). Available: http://www.dor.kaiser.org/external/news/press_releases/Kaiser_Permanente,_UCSF_Scientists_Complete_NIH-Funded_Genomics_Project_Involving_100,000_People/. Accessed 13 September 2011.

[9] Herzig SJ, Howell MD, Ngo LH, Marcantonio ER (2009) Acid-suppressive medication use and the risk for hospital-acquired pneumonia. Jama 301: 2120–2128. 19470989 10.1001/jama.2009.722

[10] Klompas M, Haney G, Church D, Lazarus R, Hou X, et al (2008) Automated identification of acute hepatitis B using electronic medical record data to facilitate public health surveillance. PLoS ONE 3: e2626 doi:10.1371/journal.pone.0002626. 18612462 10.1371/journal.pone.0002626 PMC2440348

[11] Kiyota Y, Schneeweiss S, Glynn RJ, Cannuscio CC, Avorn J, et al (2004) Accuracy of Medicare claims-based diagnosis of acute myocardial infarction: estimating positive predictive value on the basis of review of hospital records. American heart journal 148: 99–104. 15215798 10.1016/j.ahj.2004.02.013

[12] Dean BB, Lam J, Natoli JL, Butler Q, Aguilar D, et al (2009) Use of Electronic Medical Records for Health Outcomes Research: A Literature Review. Med Care Res Rev Available: http://www.ncbi.nlm.nih.gov/entrez/query.fcgi?cmd=Retrieve&db=PubMed&dopt=Citation&list_uids=19279318. 10.1177/1077558709332440 19279318

[13] Elixhauser A, Steiner C, Harris DR, Coffey RM (1998) Comorbidity measures for use with administrative data. Medical care 36: 8–27. 9431328 10.1097/00005650-199801000-00004

[14] Charlson ME, Pompei P, Ales KL, MacKenzie CR (1987) A new method of classifying prognostic comorbidity in longitudinal studies: development and validation. Journal of chronic diseases 40: 373–383. 3558716 10.1016/0021-9681(87)90171-8

[15] Li L, Chase HS, Patel CO, Friedman C, Weng C (2008) Comparing ICD9-encoded diagnoses and NLP-processed discharge summaries for clinical trials pre-screening: a case study. AMIA. Annual Symposium proceedings/AMIA Symposium 404–408. PMC2656007 18999285

[16] Elkin PL, Ruggieri AP, Brown SH, Buntrock J, Bauer BA, et al (2001) A randomized controlled trial of the accuracy of clinical record retrieval using SNOMED-RT as compared with ICD9-CM. Proceedings/AMIA. Annual Symposium 159–163. PMC2243271 11825173

[17] Ritchie MD, Denny JC, Crawford DC, Ramirez AH, Weiner JB, et al (2010) Robust replication of genotype-phenotype associations across multiple diseases in an electronic medical record. Am J Hum Genet 86: 560–572 doi:10.1016/j.ajhg.2010.03.003. 20362271 10.1016/j.ajhg.2010.03.003 PMC2850440

[18] Liao KP, Cai T, Gainer V, Goryachev S, Zeng-treitler Q, et al (2010) Electronic medical records for discovery research in rheumatoid arthritis. Arthritis Care Res (Hoboken) 62: 1120–1127 doi:10.1002/acr.20184. 20235204 10.1002/acr.20184 PMC3121049

[19] Conway M, Berg RL, Carrell D, Denny JC, Kho AN, et al (2011) Analyzing the heterogeneity and complexity of electronic health record oriented phenotyping algorithms. AMIA Annu Symp Proc 2011: 274–283. 22195079 PMC3243189

[20] Denny JC, Peterson JF, Choma NN, Xu H, Miller RA, et al (2010) Extracting timing and status descriptors for colonoscopy testing from electronic medical records. J Am Med Inform Assoc 17: 383–388 doi:10.1136/jamia.2010.004804. 20595304 10.1136/jamia.2010.004804 PMC2995656

[21] Huff SM, Rocha RA, McDonald CJ, De Moor GJ, Fiers T, et al (1998) Development of the Logical Observation Identifier Names and Codes (LOINC) vocabulary. J Am Med Inform Assoc 5: 276–292. 9609498 10.1136/jamia.1998.0050276 PMC61302

[22] Logical Observation Identifiers Names and Codes (2007). Available: http://www.regenstrief.org/medinformatics/loinc/.

[23] Kullo IJ, Ding K, Jouni H, Smith CY, Chute CG (2010) A genome-wide association study of red blood cell traits using the electronic medical record. PLoS ONE 5: e13011 doi:10.1371/journal.pone.0013011. 20927387 10.1371/journal.pone.0013011 PMC2946914

[24] Rosenbloom ST, Stead WW, Denny JC, Giuse D, Lorenzi NM, et al (2010) Generating Clinical Notes for Electronic Health Record Systems. Appl Clin Inform 1: 232–243 doi:10.4338/ACI-2010-03-RA-0019. 21031148 10.4338/ACI-2010-03-RA-0019 PMC2963994

[25] Rosenbloom ST, Denny JC, Xu H, Lorenzi N, Stead WW, et al (2011) Data from clinical notes: a perspective on the tension between structure and flexible documentation. J Am Med Inform Assoc 18: 181–186 doi:10.1136/jamia.2010.007237. 21233086 10.1136/jamia.2010.007237 PMC3116264

[26] Rasmussen LV, Peissig PL, McCarty CA, Starren J (2012) Development of an optical character recognition pipeline for handwritten form fields from an electronic health record. Journal of the American Medical Informatics Association: JAMIA 19: e90–e95 doi:10.1136/amiajnl-2011-000182. 21890871 10.1136/amiajnl-2011-000182 PMC3392858

[27] Peissig PL, Rasmussen LV, Berg RL, Linneman JG, McCarty CA, et al (2012) Importance of multi-modal approaches to effectively identify cataract cases from electronic health records. J Am Med Inform Assoc 19: 225–234 doi:10.1136/amiajnl-2011-000456. 22319176 10.1136/amiajnl-2011-000456 PMC3277618

[28] Denny JC, Spickard A, Miller RA, Schildcrout J, Darbar D, et al (2005) Identifying UMLS concepts from ECG Impressions using KnowledgeMap. AMIA. Annual Symposium proceedings/AMIA Symposium 196–200. PMC1479847 16779029

[29] Willems JL, Abreu-Lima C, Arnaud P, van Bemmel JH, Brohet C, et al (1991) The diagnostic performance of computer programs for the interpretation of electrocardiograms. The New England journal of medicine 325: 1767–1773. 1834940 10.1056/NEJM199112193252503

[30] Poon EG, Keohane CA, Yoon CS, Ditmore M, Bane A, et al (2010) Effect of bar-code technology on the safety of medication administration. N Engl J Med 362: 1698–1707 doi:10.1056/NEJMsa0907115. 20445181 10.1056/NEJMsa0907115

[31] FitzHenry F, Peterson JF, Arrieta M, Waitman LR, Schildcrout JS, et al (2007) Medication administration discrepancies persist despite electronic ordering. J Am Med Inform Assoc 14: 756–764 doi:10.1197/jamia.M2359. 17712089 10.1197/jamia.M2359 PMC2213483

[32] Denny JC, Arndt FV, Dupont WD, Neilson EG (2008) Increased hospital mortality in patients with bedside hippus. The American journal of medicine 121: 239–245. 18328309 10.1016/j.amjmed.2007.09.014

[33] Turchin A, Kolatkar NS, Grant RW, Makhni EC, Pendergrass ML, et al (2006) Using Regular Expressions to Abstract Blood Pressure and Treatment Intensification Information from the Text of Physician Notes. Journal of the American Medical Informatics Association 13: 691–695 doi:10.1197/jamia.M2078. 16929043 10.1197/jamia.M2078 PMC1656954

[34] Sager N, Lyman M, Bucknall C, Nhan N, Tick LJ (1994) Natural language processing and the representation of clinical data. J Am Med Inform Assoc 1: 142–160. 7719796 10.1136/jamia.1994.95236145 PMC116193

[35] Haug PJ, Ranum DL, Frederick PR (1990) Computerized extraction of coded findings from free-text radiologic reports. Work in progress. Radiology 174: 543–548. 2404321 10.1148/radiology.174.2.2404321

[36] Friedman C, Hripcsak G, Shablinsky I (1998) An evaluation of natural language processing methodologies. Proceedings/AMIA. Annual Symposium 855–859. PMC2232366 9929340

[37] Denny JC, Smithers JD, Miller RA, Spickard A (2003) “Understanding” medical school curriculum content using KnowledgeMap. J Am Med Inform Assoc 10: 351–362. 12668688 10.1197/jamia.M1176 PMC181986

[38] Dunham GS, Pacak MG, Pratt AW (1978) Automatic indexing of pathology data. Journal of the American Society for Information Science 29: 81–90. 10318395 10.1002/asi.4630290207

[39] Denny JC, Spickard A, Miller RA, Schildcrout J, Darbar D, et al (2005) Identifying UMLS concepts from ECG Impressions using KnowledgeMap. AMIA. Annual Symposium proceedings [electronic resource]/AMIA Symposium 196–200. PMC1479847 16779029

[40] Wang X, Hripcsak G, Markatou M, Friedman C (2009) Active computerized pharmacovigilance using natural language processing, statistics, and electronic health records: a feasibility study. J Am Med Inform Assoc 16: 328–337. 19261932 10.1197/jamia.M3028 PMC2732239

[41] Meystre SM, Haug PJ (2008) Randomized controlled trial of an automated problem list with improved sensitivity. International journal of medical informatics Available: http://www.ncbi.nlm.nih.gov/entrez/query.fcgi?cmd=Retrieve&db=PubMed&dopt=Citation&list_uids=18280787. 10.1016/j.ijmedinf.2007.12.001 18280787

[42] Xu H, Stenner SP, Doan S, Johnson KB, Waitman LR, et al (2010) MedEx: a medication information extraction system for clinical narratives. J Am Med Inform Assoc 17: 19–24 doi:10.1197/jamia.M3378. 20064797 10.1197/jamia.M3378 PMC2995636

[43] Melton GB, Hripcsak G (2005) Automated detection of adverse events using natural language processing of discharge summaries. J Am Med Inform Assoc 12: 448–457. 15802475 10.1197/jamia.M1794 PMC1174890

[44] Denny JC, Spickard A, Johnson KB, Peterson NB, Peterson JF, et al (2009) Evaluation of a method to identify and categorize section headers in clinical documents. J Am Med Inform Assoc 16: 806–815 doi:10.1197/jamia.M3037. 19717800 10.1197/jamia.M3037 PMC3002123

[45] Friedman C, Shagina L, Lussier Y, Hripcsak G (2004) Automated encoding of clinical documents based on natural language processing. J Am Med Inform Assoc 11: 392–402. 15187068 10.1197/jamia.M1552 PMC516246

[46] Zeng QT, Goryachev S, Weiss S, Sordo M, Murphy SN, et al (2006) Extracting principal diagnosis, co-morbidity and smoking status for asthma research: evaluation of a natural language processing system. BMC medical informatics and decision making 6: 30. 16872495 10.1186/1472-6947-6-30 PMC1553439

[47] Chapman WW, Bridewell W, Hanbury P, Cooper GF, Buchanan BG (2001) A simple algorithm for identifying negated findings and diseases in discharge summaries. Journal of biomedical informatics 34: 301–310. 12123149 10.1006/jbin.2001.1029

[48] Friedman C, Shagina L, Lussier Y, Hripcsak G (2004) Automated encoding of clinical documents based on natural language processing. J Am Med Inform Assoc 11: 392–402. 15187068 10.1197/jamia.M1552 PMC516246

[49] Denny JC, Miller RA, Waitman LR, Arrieta MA, Peterson JF (2009) Identifying QT prolongation from ECG impressions using a general-purpose Natural Language Processor. International journal of medical informatics 78 Suppl 1: S34–42. 18938105 10.1016/j.ijmedinf.2008.09.001 PMC2728459

[50] Savova GK, Masanz JJ, Ogren PV, Zheng J, Sohn S, et al (2010) Mayo clinical Text Analysis and Knowledge Extraction System (cTAKES): architecture, component evaluation and applications. J Am Med Inform Assoc 17: 507–513 doi:10.1136/jamia.2009.001560. 20819853 10.1136/jamia.2009.001560 PMC2995668

[51] Aronson AR, Lang F-M (2010) An overview of MetaMap: historical perspective and recent advances. J Am Med Inform Assoc 17: 229–236 doi:10.1136/jamia.2009.002733. 20442139 10.1136/jamia.2009.002733 PMC2995713

[52] Sirohi E, Peissig P (2005) Study of effect of drug lexicons on medication extraction from electronic medical records. Pac Symp Biocomput 308–318. 15759636 10.1142/9789812702456_0029

[53] Wilke RA, Berg RL, Linneman JG, Zhao C, McCarty CA, et al. (2008) Characterization of low-density lipoprotein cholesterol-lowering efficacy for atorvastatin in a population-based DNA biorepository. Basic Clin Pharmacol Toxicol 103: 354–359. doi:10.1111/j.1742-7843.2008.00291.x. 10.1111/j.1742-7843.2008.00291.x 18834356

[54] Uzuner Ö, Solti I, Cadag E (2010) Extracting medication information from clinical text. Journal of the American Medical Informatics Association 17: 514–518 doi:10.1136/jamia.2010.003947. 20819854 10.1136/jamia.2010.003947 PMC2995677

[55] McCarty CA, Nair A, Austin DM, Giampietro PF (2007) Informed consent and subject motivation to participate in a large, population-based genomics study: the Marshfield Clinic Personalized Medicine Research Project. Community Genet 10: 2–9 doi:10.1159/000096274. 17167244 10.1159/000096274

[56] NUgene Project (n.d.). Available: https://www.nugene.org/. Accessed 16 September 2012.

[57] Kaiser Permanente, UCSF Scientists Complete NIH-Funded Genomics Project Involving 100,000 People (n.d.). Available: http://www.dor.kaiser.org/external/news/press_releases/Kaiser_Permanente,_UCSF_Scientists_Complete_NIH-Funded_Genomics_Project_Involving_100,000_People/. Accessed 13 September 2011.

[58] Roden DM, Pulley JM, Basford MA, Bernard GR, Clayton EW, et al (2008) Development of a large-scale de-identified DNA biobank to enable personalized medicine. Clinical pharmacology and therapeutics 84: 362–369. 18500243 10.1038/clpt.2008.89 PMC3763939

[59] Gupta D, Saul M, Gilbertson J (2004) Evaluation of a deidentification (De-Id) software engine to share pathology reports and clinical documents for research. American journal of clinical pathology 121: 176–186. 14983930 10.1309/E6K3-3GBP-E5C2-7FYU

[60] Aberdeen J, Bayer S, Yeniterzi R, Wellner B, Clark C, et al (2010) The MITRE Identification Scrubber Toolkit: design, training, and assessment. Int J Med Inform 79: 849–859 doi:10.1016/j.ijmedinf.2010.09.007. 20951082 10.1016/j.ijmedinf.2010.09.007

[61] Uzuner O, Luo Y, Szolovits P (2007) Evaluating the state-of-the-art in automatic de-identification. J Am Med Inform Assoc 14: 550–563 doi:10.1197/jamia.M2444. 17600094 10.1197/jamia.M2444 PMC1975792

[62] Cardon LR, Palmer LJ (2003) Population stratification and spurious allelic association. Lancet 361: 598–604 doi:10.1016/S0140-6736(03)12520-2. 12598158 10.1016/S0140-6736(03)12520-2

[63] Price AL, Patterson NJ, Plenge RM, Weinblatt ME, Shadick NA, et al (2006) Principal components analysis corrects for stratification in genome-wide association studies. Nat Genet 38: 904–909 doi:10.1038/ng1847. 16862161 10.1038/ng1847

[64] Dumitrescu L, Ritchie MD, Brown-Gentry K, Pulley JM, Basford M, et al (2010) Assessing the accuracy of observer-reported ancestry in a biorepository linked to electronic medical records. Genet Med 12: 648–650 doi:10.1097/GIM.0b013e3181efe2df. 20733501 10.1097/GIM.0b013e3181efe2df PMC2952033

[65] Sohn M-W, Zhang H, Arnold N, Stroupe K, Taylor BC, et al (2006) Transition to the new race/ethnicity data collection standards in the Department of Veterans Affairs. Popul Health Metr 4: 7 doi:10.1186/1478-7954-4-7. 16824220 10.1186/1478-7954-4-7 PMC1539022

[66] Savova GK, Fan J, Ye Z, Murphy SP, Zheng J, et al (2010) Discovering peripheral arterial disease cases from radiology notes using natural language processing. AMIA Annu Symp Proc 2010: 722–726. 21347073 PMC3041293

[67] Tatonetti NP, Denny JC, Murphy SN, Fernald GH, Krishnan G, et al (2011) Detecting Drug Interactions From Adverse-Event Reports: Interaction Between Paroxetine and Pravastatin Increases Blood Glucose Levels. Clin Pharmacol Ther Available: http://www.ncbi.nlm.nih.gov/pubmed/21613990. Accessed 7 June 2011. 10.1038/clpt.2011.83 PMC3216673 21613990

[68] Rzhetsky A, Wajngurt D, Park N, Zheng T (2007) Probing genetic overlap among complex human phenotypes. Proc Natl Acad Sci USA 104: 11694–11699 doi:10.1073/pnas.0704820104. 17609372 10.1073/pnas.0704820104 PMC1906727

[69] Chen DP, Weber SC, Constantinou PS, Ferris TA, Lowe HJ, et al (2008) Novel integration of hospital electronic medical records and gene expression measurements to identify genetic markers of maturation. Pac Symp Biocomput 243–254. 18229690 PMC2716394

[70] Wood GC, Still CD, Chu X, Susek M, Erdman R, et al (2008) Association of chromosome 9p21 SNPs with cardiovascular phenotypes in morbid obesity using electronic health record data. Genomic Med 2: 33–43 doi:10.1007/s11568-008-9023-z. 18716918 10.1007/s11568-008-9023-z PMC2518660

[71] Kurreeman F, Liao K, Chibnik L, Hickey B, Stahl E, et al (2011) Genetic basis of autoantibody positive and negative rheumatoid arthritis risk in a multi-ethnic cohort derived from electronic health records. Am J Hum Genet 88: 57–69 doi:10.1016/j.ajhg.2010.12.007. 21211616 10.1016/j.ajhg.2010.12.007 PMC3014362

[72] Denny JC, Ritchie MD, Crawford DC, Schildcrout JS, Ramirez AH, et al (2010) Identification of genomic predictors of atrioventricular conduction: using electronic medical records as a tool for genome science. Circulation 122: 2016–2021 doi:10.1161/CIRCULATIONAHA.110.948828. 21041692 10.1161/CIRCULATIONAHA.110.948828 PMC2991609

[73] Crosslin DR, McDavid A, Weston N, Nelson SC, Zheng X, et al (2012) Genetic variants associated with the white blood cell count in 13,923 subjects in the eMERGE Network. Hum Genet 131: 639–652 doi:10.1007/s00439-011-1103-9. 22037903 10.1007/s00439-011-1103-9 PMC3640990

[74] Denny JC, Crawford DC, Ritchie MD, Bielinski SJ, Basford MA, et al (2011) Variants Near FOXE1 Are Associated with Hypothyroidism and Other Thyroid Conditions: Using Electronic Medical Records for Genome- and Phenome-wide Studies. Am J Hum Genet 89: 529–542 doi:10.1016/j.ajhg.2011.09.008. 21981779 10.1016/j.ajhg.2011.09.008 PMC3188836

[75] Kullo IJ, Ding K, Shameer K, McCarty CA, Jarvik GP, et al (2011) Complement receptor 1 gene variants are associated with erythrocyte sedimentation rate. Am J Hum Genet 89: 131–138 doi:10.1016/j.ajhg.2011.05.019. 21700265 10.1016/j.ajhg.2011.05.019 PMC3135803

[76] Kho AN, Hayes MG, Rasmussen-Torvik L, Pacheco JA, Thompson WK, et al (2012) Use of diverse electronic medical record systems to identify genetic risk for type 2 diabetes within a genome-wide association study. J Am Med Inform Assoc 19: 212–218 doi:10.1136/amiajnl-2011-000439. 22101970 10.1136/amiajnl-2011-000439 PMC3277617

[77] Carroll RJ, Thompson WK, Eyler AE, Mandelin AM, Cai T, et al (2012) Portability of an algorithm to identify rheumatoid arthritis in electronic health records. Journal of the American Medical Informatics Association: JAMIA 19: e162–e169 doi:10.1136/amiajnl-2011-000583. 22374935 10.1136/amiajnl-2011-000583 PMC3392871

[78] Denny JC, Kho A, Chute CG, Carrell D, Rasmussen L, et al.. (2010) Use of Electronic Medical Records for Genomic Research – Preliminary Results and Lessons from the eMERGE Network.

[79] Denny JC, Ritchie MD, Basford MA, Pulley JM, Bastarache L, et al (2010) PheWAS: demonstrating the feasibility of a phenome-wide scan to discover gene-disease associations. Bioinformatics 26: 1205–1210 doi:10.1093/bioinformatics/btq126. 20335276 10.1093/bioinformatics/btq126 PMC2859132

[80] Denny JC, Bastarache L, Crawford DC, Ritchie MD, Basford MA, et al (2010) Scanning the EMR Phenome for Gene-Disease Associations using Natural Language Processing. Proc AMIA Annu Fall Symp

[81] Scott LJ, Mohlke KL, Bonnycastle LL, Willer CJ, Li Y, et al (2007) A genome-wide association study of type 2 diabetes in Finns detects multiple susceptibility variants. Science 316: 1341–1345. 17463248 10.1126/science.1142382 PMC3214617

[82] Collins F (2009) Opportunities and challenges for the NIH–an interview with Francis Collins. Interview by Robert Steinbrook. N Engl J Med 361: 1321–1323 doi:10.1056/NEJMp0905046. 19759378 10.1056/NEJMp0905046

