# Supplementary material for: Chapter 13: Mining Electronic Health Records in the Genomics Era
Source: PLoS Comput Biol. 2012 Dec 27;8(12):e1002823. doi: 10.1371/journal.pcbi.1002823 (PMC3531280; doi:10.1371/journal.pcbi.1002823)
Supplement: Text S1 — Answers to Exercises. (DOCX) [file pcbi.1002823.s001.docx]

Exercises (with Answers)

1. Compare and contrast the basic types of data available in an Electronic Health Records (EHR) that are useful for mining genetic data. What are some of the strengths and drawbacks of each type of data?

**Answer**: See section 2, “Classes of Data available in EHRs”, and Table 1. There is a tension between ease of querying, accuracy of data, and sensitivity of analysis when mining EHR data for clinical or genomic research. For many diseases, ICD9s may have good sensitivity and provide an adequate level of diseases specificity (e.g., myocardial infarction) but for rarer diseases, other sources of information may be needed (e.g., ST-elevation myocardial infarction, non-alchoholic steatohepatitis – neither of which have a specific ICD9 code).

1. Explain what a phenotype algorithm is and why it is necessary. For example, how can use of natural language processing improve upon use of billing codes alone?

**Answer**: See section 6, “Phenotype-driven discovery in EHRs”. Phenotype algorithms are typically combinations billing codes, laboratory (and other) tests, medication exposures, and/or natural language processing or text queries. Algorithms can also use machine learning methods operating on large feature sets. Compared to simply querying a single mode of data, phenotype algorithms can improve sensitivity, specificity, or both for discovery a given phenotype. Reasons why multi-modal algorithms are needed include: lack of an available billing code or other structured reference, need to capture historical data, differentiating between multiple potentially overlapping conditions, and capturing rare events. NLP can add to billing codes by allowing capture of more granular phenotypes (e.g., ST-elevation myocardial infarction) and serve to “confirm” a billing code via clinical documentation or detect exclusions (e.g., patients with a family history of certain diseases).

1. Select a clinical disease and design a phenotype algorithm for it.

**Answer**: A general approach, after selection of a disease to study, would be to identify relevant feature for the diagnosis, billing and treatment for the disease. Often, these can be combined in an AND-OR-NOT Boolean logic.

Consider Type 2 diabetes. To identify billing codes, a number of public websites are available (e.g., <http://icd9cm.chrisendres.com/>). We will include the codes for Type 2 diabetes. Then, consulting general medical references for diagnostic criteria for diabetes and medications, we see that diabetes is defined as a random glucose > 200 mg/dl, fasting glucose > 126 mg/dl, or hemoglobin A1c > 6.5%. However, since EHR-derived glucose values typically do not specify fasting or not (and even when they do, are often inaccurate), the second laboratory criteria is not that helpful. Thus, we will use the A1c and random glucose cutoffs (>200 mg/dl). Type 1 diabetes typically includes long and short acting insulin, and rarely includes oral medications. The mainstay of type 2 diabetes is oral medications, with addition of insulin typically as the disease progresses and endogenous insulin production decreases, so we include a list of oral medications specific to type 2 diabetes in our algorithm. Finally, we may wish to exclude patients who have ever had a type 1 diabetes code, and patients who only had diabetes codes (or medications) during pregnancy. After proposing such an algorithm, it is important to run the algorithm and review cases to determine if it achieves a sufficient positive predictive value and an adequate number of cases. In some cases, one may wish to formally evaluate sensitivity (recall) as well.

For a list of some current EHR phenotype algorithms (including algorithms for type 2 diabetes), please see <http://PheKB.org>.

1. How might a phenotype algorithm be different for a very rare disease (e.g., prion diseases) vs. a more common one (e.g., Type 2 diabetes)? How would a phenotype algorithm be different for a physical exam finding (e.g., hippus or a murmur) vs. a disease?

**Answer**: See Section 6.2. For a very rare disease (such as a prion disease), one is likely to try to retrieve every possible case, and worry less for the algorithm’s positive predictive value. In addition, for rare diseases, one is more likely to use a combination of billing codes, natural language processing, and/or lab tests, potentially unioned together. For a physical exam finding like hippus, one would need to perform a full text search of the EHR (there is not corresponding billing code, for instance). For specific heart murmurs, one may want to restrict to notes written by cardiologists, or instead use echocardiograms that specifically interrogate valvular anatomy instead of relying on the physical exam itself.

1. Describe the differences between a DNA biobank linked to an EHR and one collected as part of a non-EHR research cohort. What are the advantages and disadvantages of a de-identified DNA biobank vs. an identified DNA biobank (either linked to an EHR or not).

**Answer**: See Section 4 and 5. One of the key advantages of an EHR-associated biobanks is that they collect a diverse set of longitudinal data cheaply, and these observations are not biased toward a particular phenotype or study. However, this data can be fragmented between different healthcare systems, lack in accuracy, and may have biases toward conditions and findings that reimburse. Moreover, the presumed diagnosis may not be validated to the same degree as it would in a research cohort (e.g., a physician may treat empirically for a given diagnosis without performing the confirmatory tests). Certain study measurements, such as formal rating scales for rheumatoid arthritis severity or depression, common in research studies, are noticeably lacking in EHRs. Finally, follow-up in EHRs may be more sporadic than in clinical trials or observational cohorts.

Identified biobanks have an advantage over de-identified biobanks in that they allow recontact of participants, but they may accrue patients more slowly.

1. It is often harder to create algorithms to find drug-response phenotypes (such as adverse drug events) than for a chronic disease. Give several reasons why this might be.

**Answer**: Drug-response phenotypes typically require recognition of several phenotypes, sequenced in order, to achieve accuracy. For instance, consider evaluation of clopidogrel efficacy to prevent major cardiovascular events following placement of a cardiac stent: the patients need to have received a stent, by placed on (and be compliant with) clopidogrel therapy, and then have subsequent measurement of whether the patient had a subsequent adverse event (myocardial infraction, stroke, revascularization, or death) while on clopidogrel. Subsequent events may occur at an outside hospital, leading to falsely representing a case as a control. Compliance issues may lead to falsely include an individual in a study when they were not actually exposure to the medications. Recognition of each of these elements has inaccuracies, whose errors can be additive.
